# Barriers and Facilitators to Scaling Up the Non-Pneumatic Anti-Shock Garment for Treating Obstetric Hemorrhage: A Qualitative Study

**DOI:** 10.1371/journal.pone.0150739

**Published:** 2016-03-03

**Authors:** Keely Jordan, Elizabeth Butrick, Gavin Yamey, Suellen Miller

**Affiliations:** 1 College of Global Public Health, New York University, New York, New York, United States of America; 2 Private Sector Healthcare Initiative, Global Health Group, University of California, San Francisco, United States of America; 3 Duke Global Health Institute, Duke University, Durham, North Carolina, United States of America; 4 Safe Motherhood Program, Bixby Center for Global Reproductive Health, University of California, San Francisco, United States of America; Banaras Hindu University, INDIA

## Abstract

**Background:**

Obstetric hemorrhage (OH), which includes hemorrhage from multiple etiologies during pregnancy, childbirth, or postpartum, is the leading cause of maternal mortality and accounts for one-quarter of global maternal deaths. The Non-pneumatic Anti-Shock Garment (NASG) is a first-aid device for obstetric hemorrhage that can be applied for post-partum/post miscarriage and for ectopic pregnancies to buy time for a woman to reach a health care facility for definitive treatment. Despite successful field trials, and endorsement by safe motherhood organizations and the World Health Organization (WHO), scale-up has been slow in some countries. This qualitative study explores contextual factors affecting uptake.

**Methods:**

From March 2013 to April 2013, we conducted 13 key informant interviews across four countries with a large burden of maternal mortality that had achieved varying success in scaling up the NASG: Ethiopia, India, Nigeria, and Zimbabwe. These key informants were health providers or program specialists working with the NASG. We applied a health policy analysis framework to organize the results. The framework has five domains: attributes of the intervention, attributes of the implementers, delivery strategy, attributes of the adopting community, the socio-political context, and the research context.

**Results:**

The interviews from our study found that relevant facilitators for scale-up are the simplicity of the device, local and international champions, well-developed training sessions, recommendations by WHO and the International Federation of Gynecology and Obstetrics, and dissemination of NASG clinical trial results. Barriers to scaling up the NASG included limited health infrastructure, relatively high upfront cost of the NASG, initial resistance by providers and policy makers, lack of in-country champions or policy makers advocating for NASG implementation, inadequate return and exchange programs, and lack of political will.

**Conclusions:**

There was a continuum of uptake ranging in both speed and scale. Ethiopia while not the first country to use the NASG has the most rapid scale-up, followed by Nigeria, then India, and finally Zimbabwe. Increasing the coverage of the NASG will require collaboration with local NASG champions, greater NASG awareness among clinicians and policymakers, as well as stronger political will and advocacy.

## Introduction

About 290,000 women die each year from complications of pregnancy and childbirth [1]. The most common cause of death is post-partum hemorrhage (PPH) [2]. In low-resource settings, death from PPH is often the result of institutional, environmental, cultural and social barriers to receiving skilled care, which involves prevention, diagnosis and treatment of PPH [2]. The international standard for PPH prevention is active management of the third stage of labor (AMTSL), which includes the administration of uterotonic medicines within five minutes of birth and uterine massage [3]. Treatment of PPH at the primary care level also includes uterotonics. If bleeding does not stop, referral to higher levels for blood transfusions and/or surgery may be necessary [3]. Many causes of obstetric hemorrhage, however, do not respond to uterotonics. In addition, in low-resource settings conventional uterotonics may not be available and women may experience long delays before treatment [4].

There are three delays that contribute to maternal mortality: (1) the delay in decision-making to seek medical care; (2) the delay in reaching a facility staffed and stocked for obstetric emergencies; and (3) the delay in receiving quality emergency care [5,6]. A first aid device, the Non-pneumatic Anti-Shock Garment (NASG), can be applied to a woman with PPH in order to stabilize the woman during the second and third delays [4,5,6]. The NASG, made of neoprene and Velcro^™^, compresses the lower body with nine articulated segments closed sequentially and tightly around the legs, pelvis and abdomen. Tamponade of abdominal, pelvic, and uterine vessels reduces blood loss and reverses shock [4]. The NASG is appropriate for use in developing countries due to its simple application and relatively low cost. The device costs USD 57.50 from one manufacturer in Hong Kong, and can be re-used at least 72 times. Two cost-effectiveness analyses have found the NASG to be cost-beneficial or extremely cost effective [7,8]. After training, any trained birth attendant can rapidly apply the NASG on a hemorrhaging woman and with the bleeding under control she can be safely transported to a facility, or between facilities, for definitive care [8]. In low-resource settings, even at the highest-level referral hospital, there is often a long delay in receiving blood transfusions or surgery, therefore the NASG can be used in facilities where women often wait hours for blood and surgery [9]. The NASG can be easily cleaned with a dilute, 0.01% bleach solution, washed with detergent by hand or in a washing machine, folded and reused. However, if the NASG is placed on a woman at a lower level facility, and the woman is transferred to a referral facility, the same NASG must remain on the woman until she is stable. Therefore, in low income settings, if a woman is transported from a lower level facility to a higher-level facility, a return and exchange program is necessary. In higher income settings, such as the US or UK, a single use policy could be considered.

Quasi-experimental trials of the NASG in Egypt and Nigeria, conducted by the Safe Motherhood Program of the Bixby Center of Global Reproductive Health at the University of California San Francisco (UCSF) and international colleagues, found that use of the device was associated with statistically significant reductions in median measured blood loss, maternal mortality, severe morbidity (end-organ failure), and emergency hysterectomies [10,11,12]. An external systematic review of NASG studies was recently published and found that where samples were pooled there is a statistically significant reduction in maternal mortality (RR = 0.52, 95% CI 0.36–0.77) and a statistically significant reduction in any adverse outcome (combination of mortality and severe morbidity) (RR = 0.31, 95% CI 0.17–0.59) [13]. A 70% reduction in mortality/near miss is clinically critical.

A cluster randomized control trial, also conducted by UCSF and colleagues in Zambia and Zimbabwe, of applying the NASG at primary care centers before transporting women to referral hospitals (where control arm women received the NASG) found a non-significant 46% reduction in the odds of mortality (odds ratio 0.54, 95% confidence interval 0.14–2.05, p = 0.37). Women who received the NASGs recovered from shock significantly faster (hazard ratio 1.25, 95% CI 1.02–1.52, p = 0.03) [14]. While not statistically significant the trials demonstrated a clinically important reduction in mortality and a statistically significant more rapid recovery from shock with earlier application. The trial authors concluded that: “As there are no other tools for shock management outside of referral facilities, and no safety issues found, consideration of NASGs as a temporizing measure during delays may be warranted” [14].

In 2012 the NASG is recommended by (a) the World Health Organization in its *Recommendations for the Prevention and Treatment of Postpartum Hemorrhage*; (b) the International Federation of Gynecology and Obstetrics (FIGO) in its *Guidelines of Best Practices for the Prevention and Treatment of Postpartum Hemorrhage in Low-resource Settings*; and (c) the Global Library of Women’s Medicine in their *Guidelines for Immediate Action in Treating Postpartum Hemorrhage* [15,16,17]. Nevertheless, the adoption and uptake of the NASG was highly variable across different countries. We therefore conducted a study to examine the barriers and facilitators to scaling up coverage with the NASG.

## Methods

We conducted a qualitative study from March 2013 through April 2013 involving in-depth individual (one-on-one) interviews with 13 key informants across four countries, using a purposive and snowballing sampling. We initially interviewed a set of policy makers and health care providers who had used the NASG, as well as safe motherhood program specialists. These informants were identified by the UCSF Safe Motherhood team based on their known involvement with the NASG and purposively sampled based on their relevant expertise or experience. These contacts then referred us to other key informants and stakeholders, who were also country specific experts.

Interviews were conducted by one of the authors (KJ) in English, as all potential interviewees had a working fluency, either over the phone or Skype from the US to the respective countries (see S1 Appendix for the interview guide and S2 Appendix for a profile of the key informants). Interviews lasted one and a half hours on average and were recorded using an audio device recorder and subsequently transcribed. Follow-up questions or clarifications were then conducted via email with the interviewees when necessary.

The four countries in this study were selected partly as a convenience sample, and partly to reflect a range of countries that have shown varying success in scaling up the NASG. These four countries were all involved with UCSF trials and/or implementation projects for which UCSF supplied technical assistance. They all have a large burden of maternal mortality due to obstetric hemorrhage. They have differing experiences in their adoption and scale-up of the NASG. The four countries were Ethiopia (2 interviews), India (3 interviews), Nigeria (4 interviews), and Zimbabwe (4 interviews). Each country is at a varying stage in the implementation process- starting with the introduction of the garment through trials and/or implementation projects (Zimbabwe), followed by local incorporation of the device into protocol (India), then country wide support and dissemination (Nigeria and Ethiopia). Table 1 summarizes the recent trends in maternal mortality ratio, the proportion of maternal deaths due to PPH, and the scale-up of the NASG to date in each of these countries.

**Table 1:** Characteristics of the study countries: trends in the maternal mortality ratio (MMR) proportion of maternal deaths due to PPH, and scale-up of the NASG.

Country	Recent trends in MMR	Burden of PPH	Status of NASG uptake
Ethiopia	MMR decreased between 2005 and 2013 from 740 to 420 deaths per 100,000 live births [1]	Ministry of Health estimates that 10% of maternal deaths are to PPH [18]	Clinton Health Access Initiative introduced NASG in Oromia and Tigray provinces as pilot project in June 2011 [19].
		Unpublished documents suggest that PPH is associated with 25–50% of all maternal deaths [18]	Pilot was followed by expansion nationwide to 110 hospitals (out of 130 total) and 649 health centers (out of 2661 total) and by training of staff in referral hospitals in all 9 regions by April 2014 [19].
			Scale-up strategy based on strong political support and timing.
India	MMR decreased between 2005 and 2013 from 280 to 190 deaths per 100,000 live births [1]	PPH is associated with 30% of all maternal deaths [20]	Pathfinder International (an international NGO) implemented NASG project from 2007–2012—it was approved for use only in the NGO’s project facilities in Rajasthan and Maharashtra states [21].
			Later the project added the states of Bihar, Uttar Pradesh, Odisha (formally Orissa), and Assam [21].
			Tamil Nadu used the NASG in government facilities and did not participate in the Pathfinder International project outside of acquiring the NASGs and receiving the initial training package.
			In 2013 there were about 3,700 NASGs in the Pathfinder International project areas, with an additional 1,200 NASGs in Tamil Nadu alone [21].
			Scale-up strategy differs by state, no country level coordinated plan.
Nigeria	MMR decreased between 2005 and 2013 from 740 to 560 in 2013 [1]	PPH is associated with 30–40% of all maternal deaths [22]	First country to implement the NASG
			Initial UCSF, in collaboration with University College Hospital Ibadan and 14 other facilities, NASG trials, (2003–2007) included Nigeria; a 2008 meeting to disseminate the trial results served as the kick-off for an NASG implementation project led by Pathfinder International [23].
			The Pathfinder International project expanded implementation from 31 facilities in four states in the first year (2007) to 60 health facilities in 7 states by 2014 [23].
			As of 2013, The Pathfinder International project has scaled up to over 5,000 NASGs in circulation in 22 of 36 states [23].
			Scale-up strategy heavily focused on in-country champions.
Zimbabwe	MMR increased by 28% from 1990 to 2010, with a 2010 average MMR of 570 deaths per 100,000 live births [24]	PPH is associated with 34% of maternal deaths [24]	Two referral hospitals and 12 city council-run primary health care maternity clinics in Harare participated in the 2007–2012 UCSF cluster randomized trial of the NASG, in collaboration with University Teaching Hospital (UTH), Lusaka Zambia; U Zimbabwe-UCSF Reproductive Health Research Collaborative and U ZIM, Harare, at the clinic level (before referral to a tertiary hospital) [14]. Those results were released in a national dissemination meeting.
			In 2013, The Ministry of Health stated that it would add the NASG to the government’s essential devices list (NASG Project Coordinator—personal communication).
			Premier Service Medical Investments (PSMI)- a private medical services provision group- is implementing the NASG in Harare and Bulawayo (NASG Project Coordinator—personal communication, September 2014).
			Scale-up strategy still currently hinges on in-country trials.

We used a semi-structured interview guide, shown in S1 Appendix. We designed the questions to explore the success of the NASG scale-up in recent years, barriers to use, and facilitating factors for future expansion. The guide was adapted following initial interviews in order to more clearly address the themes in question. We recorded the interviews using an audio device recorder and subsequently one of the authors (KJ) transcribed them. Two authors (KJ, EB) coded the transcriptions independently using a code book generated by three of the authors (KJ, EB, and SM) as shown in S3 Appendix. No coding software was used. Once each transcription was coded, authors KJ and EB compared the results of their independent coding. Where discrepancies arose, a third author (SM) was brought in to review the results, which were then discussed by three authors (KJ, EB, SM) until a consensus was reached. We organized emerging themes using the Scaling Up Global Health Interventions Framework previously developed and published by the 4^th^ author (GY) [25]. This framework categorizes different components of the scale-up processes as follows:

- attributes of the specific tool or service being scaled up,
- attributes of the implementers,
- the chosen delivery strategy,
- attributes of the “adopting” community,
- the socio-political context, and
- the research context [23].

### Ethical Statement

The interviewees were health care providers and program specialists. No patients or vulnerable populations were interviewed. The University of California, San Francisco’s institutional review board, the Committee on Human Research (CHR), certified the study as exempt (IRB number 13–10606). The goals and objectives of the study were described during the recorded phone sessions to each key informant and verbal consent was obtained before every interview using a CHR approved verbal consent script. Written consent was not sought due to geographical limitations and the exempt nature of the study. To protect the anonymity of the key informants, any identifying information was removed from the paper.

## Results

### Attributes of the NASG

All13 key informants (KIs) discussed individual attributes of the NASG as being both facilitators and barriers to scale-up of the NASG. The simplicity of the device was described by six informants across all four countries as easy to use, easy to clean and care for, and effective in saving lives (KIs 2, 3, 5, 6, 9, 12). The cost of the NASG in 2012 was cited as a major initial concern by KIs in Ethiopia, India, and Zimbabwe (KI 1, 5, 11, 13), although one KI (KI 12) argued that the benefits of the NASG outweighed its costs:

*“I doubt that many people could be opposed to an intervention that saves lives*. *If you are saving lives and spending a little bit more than you would have*, *it is necessary and worth it*.*”*

KI 12

### Attributes of the implementers

KIs in Ethiopia (KIs 1, 2) attributed the rapid implementation of the device to the work of task forces, provider support, and in-country champions.

*“We even formed a task force and we updated them about the device and we announced that we secured government approval so there was a lot of advocacy*, *there was a lot of going from one office to another and knocking on a door that needs to be knocked to get approval for the device*.*”*

KI 1

Key informants in Nigeria highlighted the importance of partnerships between NGOs, professional associations, multi-lateral organizations/donors and government agencies (KIs 7,9). It is through these partnerships, they argued, that training sessions were held across the country for doctors, nurses, and midwives (KIs 7, 9). The implementers created a multi-pronged training approach, including provider focus groups, in order to overcome resistance; such diverse and widespread influential champions are a key component to the depth and penetration of the NASG in Nigeria (KI 8).

*“We have certainly tried to bring in different models*, *and the community*, *and mobilization strategies to talk about how we think health care providers should be empowered and trained to address some of these barriers*.*”*

KI 8

In India and Zimbabwe, however, key informants expressed concern that national support was going to take time and acceptance by the government was essential for scale-up (KIs 3, 5, 12, and 13).

*“Every effort should be made to convince NRHM (National Rural Health Mission) Officials for the approval of usage of the NASG and only they can make the NASG available and accessible to all maternity staff in India*.*”*

KI 3

### Delivery strategy

In Ethiopia, a systematic delivery system was key to fast expansion of NASG coverage (KIs 1, 2). After the initial pilot project, additional hospitals and then lower level facilities were trained and stocked with the device quickly (KI 2). This integration insured that if a patient arrived at a higher-level facility, maternity providers were familiar with proper techniques for care and management of patients in the NASG (KI 1). Nigeria also started training at the tertiary level and worked down to lower levels but at a slower pace over time (KI 7). More recent delivery efforts in Nigeria have been widespread and the training sessions have begun to include all providers interested regardless of what level care facility the provider comes from (KI 7). The clinical trial of the NASG in Zimbabwe concluded in June 2013 and all four key informants cautioned that the delivery strategy must be strategic in order to have successful expansion. KI 13 suggested following the Ethiopia and Nigeria examples to start at the tertiary level and systematically work down to lower level facilities in order to prevent confusion.

*“So it will be easy for someone [at the central hospital] to receive someone in the garment because they know exactly what to do*. *Rather than if we started at the primary and someone appeared at the central hospital in this blue suit without knowing what do it*. *It would be chaos*.*”*

KI 13

All three key informants in India (KIs 3, 4, 5) expressed concern that it will be difficult to deliver the NASG widely across the country, given that it has very large rural areas. Key informants were also concerned about exchange and return policies, i.e. how the NASG on a woman transported to a higher level facility would get returned to its original facility (KIs 4, 5):

*“It’s a big challenge*. *We had a mandate that if you give one you send it back but then you need the supplies and accountability*.*”*

KI 5

### Attributes of the adopting community

All 13 key informants discussed the high rate of maternal mortality in their country, with PPH being the leading cause. There is a recognized unmet need for the NASG in each country even though the NASG does not address the social, economic, and gender barriers to receiving care, most key informants identified their community needs, the long delays during transportation to a care facility and in receiving definitive care at referral facilities, as facilitators to scale-up (KIs 1, 3, 4, 5, 6, 7, 8, 9, 11, 12, 13).

*“In facilities like health centers there is no access to blood transfusion*, *so most of the time if there is severe bleeding they administer oxytocin and refer the mother to the hospital so that she gets a blood transfusion*. *But we know that oxytocin will not be effective for all kinds of hemorrhage…There is a huge challenge going to the health center*. *And after the health center there is another huge challenge going to the hospital*.*”*

KI 1

Ethiopia, India, Nigeria and Zimbabwe all have understaffed and overstretched health facilities as well as inadequate roads, infrastructure, and access to ambulances (KIs 1, 4, 5, 8, 9, 12, 13). The lack of blood and surgical supplies needed for definite care is so extreme in all four countries that the NASG was viewed as having a specific, critical, life-saving role in the public health sector and the health care provider communities seem eager to adopt the intervention (KIs 1, 4, 8, 9, 12, 13):

*“Yet the fact that new medical options have been so quickly integrated into treatment plans here is clear demonstration of how badly they were*, *and are*, *needed”*

KI 8

### The socio-political context

Key informants in Ethiopia argued that the introduction of the NASG was good timing in regards to strong leadership within the Ministry of Health (KIs 1, 2).

*“How we were able to introduce it was the idea came at a very good time when we were designing the program*, *so it was really good timing*. *We were looking for innovative technologies that had high impact*.*”*

KI 1

In Zimbabwe, in contrast, one KI felt that the uncertainty of the country’s political climate at the time might work against NASG implementation (KI 10).

Key informants in all four countries, recognized that implementation of a policy is a process that needs continual support, which includes attention, resources, and political backing (KIs 1, 4, 7, 9, 13). Both Nigeria key informant 7 and Zimbabwe key informants 10 and 13 expressed that national political support is necessary to increase availability.

*“The problem isn’t the number of garments but rather the political will to make it available for everybody*.*”*

KI 7

### The research context

The research context varied widely in the four countries. All four KIs in Nigeria felt that the strongest facilitator for implementation was the research team in country championing for the NASG (KIs 6–9). Ethiopia, however, worked independently of UCSF’s large ongoing research and implementation efforts due to financial constraints, but relied on previous efficacy trial results (KI 2). The government did not require national level efficacy data, nor did the government require WHO approval.

In contrast one potential barrier to country-wide dissemination in India, argued one KI, was the lack of country-specific efficacy trials, which some individuals in the national government have said are needed in order to gain acceptance (KI 5).

*“For India we need to have clinical trials and generate our own data*. *You see it is a new technology and it is being used on pregnant woman so it needs a lot of approvals…then the Indian government will say OK*.*”*

KI 5

All three KIs in India discussed the unique case of the state of Tamil Nadu, which took the initiative of introducing the NASG into the public health facilities for state-wide use, despite the lack of India specific data (KIs 3,4,5).

Zimbabwe had its clinical trial dissemination meetings in 2013 and is planning on scaling-up (KIs 12, 13). The NASG is being used at Mbuya Nehanda Maternity and a log of cases was maintained after the trial.

*“It is also important here to have and document case studies where a woman whose life has been saved by that intervention*, *even one life saved by an intervention is very important*. *And those case studies need to be documented and presented to the providers*.*”*

KI 13

## Discussion

Across four countries that were at different stages in the NASG implementation process, several key themes emerged. Due to the small sample size these results are preliminary and not generalizable but give insight into the barriers and facilitators to scale-up. Using Yamey’s framework on scaling up, these factors can usefully be organized into the characteristics of the health tool or service and of the implementers, the chosen delivery strategy, the attributes of the community adopting the tool/service, and the background context (the socio-political and research context) [25].

The interviews from our study found that relevant *facilitators to scaling up the NASG* are the simplicity of the device; its potential to save lives; the existence of strong partnerships between NGOs, professional associations, multi-lateral organizations/donors and government agencies (such partnerships can help to support advocacy); the presence of strong local and national champions for the device; well-developed training sessions on how the device is used; a clear WHO recommendation that the device should be adopted; and successful national trial results. Ethiopia followed by Nigeria and the Indian State of Tamil Nadu have had the most successful implementation of the NASG to date. The interviews implied the strongest forces driving their scale-up have been a clear delivery and training system as well as good political timing to push through scale-up. These overarching facilitators to successful implementation demonstrate the importance of a strategic scale-up plan.

The interviews from our study also found *barriers to scaling up the NASG*: the relatively high upfront cost; lack of government support for its implementation; lack of a clear delivery plan, which is strongly tied to government structure and political will, and a plan for return and exchange; difficulties in reaching large, remote rural areas; initial resistance by some health care providers; weak health systems (including poor health infrastructure and staff shortages); weak political leadership and lack of political will; and a lack of local efficacy trials on the NASG. The two countries that have had the slowest uptake, India and Zimbabwe, have most notably struggled with government support for the NASG implementation, and, in the case of India, local efficacy trials on the NASG. Successful, wide-scale implementation in Ethiopia overcame these barriers with strong in-country champions and reliance on regional efficacy data. These country specific barriers highlight that based on the interviewees experiences successful implementation has to be context specific.

### Strengths and weaknesses of the study

To the best of our knowledge, this is the first study that used in-depth individual interviews to explore factors that can impede or support the scale-up of the NASG across multiple countries. The inclusion of four different countries that are on different trajectories in their pathway to NASG scale-up allowed us to compare and contrast country experiences—which in turn helped to point to the barriers and facilitating factors in scaling up. A major strength of the study is that many of the key informants had expertise in working with the NASG; their views reflected their pragmatic, “real world” experience in implementing or researching NASG scale-up processes in a variety of settings. Two of the authors of this study [SM, EB] were involved in previous clinical trials of the NASG, which meant that our study group had good access to “high value” key informants.

Nevertheless, our study also had a number of weaknesses. Through our method of purposive sampling and snowballing, we invited a total of 45 people to be interviewed for this study, but only 13 responded and agreed to participate. Though there were many recurring themes in the responses we cannot be sure if data saturation was reached or how representative the interviewees were at the country level due to the lack of additional willing participants. Also, all 13 interviewees have a similar profile as academicians/program implementers /clinicians and therefore may not be familiar with important aspects of scale-up such as manufacturing and distribution of health commodities. This in turn may have introduced bias into the study, in that the 13 KIs may have had different views about the NASG than those who declined to be interviewed. For example, the 13 KIs may have had a more positive experience in their efforts to scale-up the device, or they may have had more positive views on the device itself. This potential bias could be overcome through conducting further research with a larger number of KIs with a broad set of views about the NASG, including those who oppose its scale-up. In order to get a more complete view additional interviews should be conducted with opponents to implementation and scale-up. In addition, while all of the transcripts were coded independently by two authors (KJ, EB), all 13 of the interviews were conducted by only a single author who had only recently been trained in qualitative methods and was associated with being a UCSF staff while UCSF was involved in the NASG trials and implementation projects (KJ). This may have influenced the interviewee’s responses or the interpretations of the findings swaying them towards a favorable view of the NASG. Finally, these interviews were conducted in 2013, with a new medical device situations can change rapidly, there are currently more countries implementing the NASG, some at scale, and these countries should be involved in any future research.

### Implications of the findings

Even with the low response rate we feel the study’s findings have important implications for both health providers and makers. Initial resistance among providers is usually the result of lack of exposure to a new health technology [26]. Since the complex process of diffusion of an innovation relies heavily on human capital, pre-service and in-service trainings on how to use the NASG for nurses, midwives, and doctors are needed to increase awareness and promote correct use [27]. Working in collaboration with provider/professional associations will also help to overcome initial resistance. In terms of implications, the NASG has already gained acceptance from expert groups, donors, and many international agencies, such as Pathfinder International, the Clinton Health Access Initiative (CHAI), *PATH*, *John Snow*, *Inc*., *Ifakara Health Institute*, *International Committee of the Red Cross*, *International Federation of Gynecologists and Obstetricians*, *Global Library of Women’s Medicine*, *and the WHO*. However, more widespread national political commitment is needed for countrywide adoption. Local champions from the health, development, and gender sectors could advance awareness and support from policy makers, giving a voice to the poor, marginalized women whose lives are potentially affected by this intervention. We are confident that the findings are useful for health providers and policy makers because of their consistency with other studies though we recognize that more interviews could reveal additional and possibly valuable points of view.

Previous research by Shiffman and Smith confirms that such public support for a policy by a cohesive network of actors is one of four important factors in getting a health issue on the agenda [28]. Therefore advocacy groups working on the NASG should highlight the large burden of obstetric hemorrhage and the role of the device in managing this life-threatening condition, and they should take advantage of policy windows—specific moments in time that make it more likely that an issue can be placed on the agenda. One example of a policy window was the adoption by United Nations member states of a new set of Sustainable Development Goals (SDGs), one of which (SDG3) is devoted to health. One of the targets of SDG3 is to reduce the global maternal mortality ratio to below 70 per 100,000 live births, a goal that will certainly require scaled up services to prevent and treat obstetric hemorrhage.

### Relationship of the findings to previous studies

Consistent with previous work on the NASG and new technology introduction, this paper finds that its use is influenced by the setting and backgrounds of the providers involved. We found that initial resistance to the NASG was usually overcome through trainings and after witnessing real cases with the NASG, similar to what Berdichevsky et al. term “doubting” [29].

This paper also found supporting data for many of the recommendations made in the May 2013 NASG Policy Brief presented by the Safe Motherhood Program of the Bixby Center for Global Reproductive Health [30].

### Unanswered questions and future research

While many factors can be controlled for and anticipated, the role of in-country champions and the timing of policy windows of opportunity seem to be a key difference influencing the continuum of uptake between countries. Strong political will and influential policy champions coupled with a policy window accelerated acceptance and scale-up, while the lack of these, despite similar country needs, facilitators and barriers appear to have slowed down uptake. Future research on the role of political timing to push NASG scale-up, specifically why it was so successful and timely in Ethiopia and the Indian state of Tamil Nadu, would shed light on some of these unanswered questions. It appears that in those places where dissemination has slowed, there was a lack of a clear scale-up plan. Perhaps focus groups involving health care providers, health clinics and hospitals could be a useful method to explain the interplay observed between grass root level demand and organization scale-up to better understand how these dynamics affect scale-up.

## Conclusion

Based on the interviews conducted there appears to be a continuum of uptake ranging in both speed and scale from Ethiopia to Nigeria, to India, to Zimbabwe. Our study has found four preliminary key messages that with further research can potentially help to guide successful NASG implementation and scale-up. First, the initial step to implementing the NASG is assessing the maternal health needs of the country and examining the existing health system in order to determine appropriate levels of integration. A clear country specific plan seems to be integral to successful and timely scale-up. Second, it is essential to identify and work with local NASG champions. In collaboration with advocacy groups, champions can gain political attention and priority for the intervention. Third, provider acceptance and adoption is important to scale-up. Initial resistance to a new technology is a common barrier and therefore integration of the device may be streamlined through pre-service and in-service trainings. Finally, strategic timing and political support is integral to wide scale fast implementation.

## Supporting Information

S1 Appendix Interview Guide for Key Informant Interviews. (DOCX)

S2 Appendix Basic Demographic Information on Each Key Informant. (DOCX)

S3 Appendix Code Book. (DOCX)
