# Supplementary material for: Barriers and Facilitators to Scaling Up the Non-Pneumatic Anti-Shock Garment for Treating Obstetric Hemorrhage: A Qualitative Study
Source: PLoS One. 2016 Mar 3;11(3):e0150739. doi: 10.1371/journal.pone.0150739 (PMC4777561; doi:10.1371/journal.pone.0150739)
Supplement: S1 Appendix — (DOCX) [file pone.0150739.s001.docx]

**S1 Appendix.** **Interview Guide for Key Informant Interviews**

1. How big of a role is obstetric hemorrhage in maternal mortality in your country?

2. We often discuss maternal mortality in terms of the delays framework, in your opinion, what proportion of obstetric hemorrhage in your country is due to a delay in recognizing a complication at a community or primary care level?

-Delay in deciding to seek medical care?

-Delay in reaching care facility?

-Delay in receiving adequate care once the woman reaches a tertiary care center?

-For example, how long do you think it takes for a woman to get blood once she is at a referral center? To get surgery if she needs surgery?

3. Are there sub-populations with higher rates of obstetric hemorrhage cases?

-Such as: rural compared to urban? Different ethnicities? Different religions? Lower socio-economic status?

-If yes, might these sub-populations be less likely to have access to a safe delivery?

4. Is there a national post-partum hemorrhage strategy in place?

-If so, at what stages of the strategy are improvements needed?

5. What is your experience with the non-pneumatic anti-shock garment- the NASG?

(If limited exposure to the NASG give brief explanation: *The Non-Pneumatic Anti-Shock Garment (NASG) is a low-technology first-aid device that may assist women to survive delays in transport and while awaiting definitive treatment. The NASG, made of neoprene and Velcro ^TM^, compresses the lower body with nine articulated segments closed sequentially and tightly around the legs, pelvis and abdomen. A foam ball in the abdominal segment increases compression. Circumferential compression reduces vascular volume under the compressed areas, while expanding central circulation and increasing preload, peripheral resistance and cardiac output. Tamponade of abdominal, pelvic, and uterine vessels reduces blood loss.)*

6. Please tell me what you know about how the NASG was first introduced in your country – who introduced it? Where? How/when did you first hear about it?

7. Please describe how the NASG has been incorporated into the public health system. What level is it being used and how widely?

8. How many NASGs are in use throughout the country?

-Which states/provinces are using the NASG?

-Why are some states using it and others not? Who decides a state will use it? Is it decided at a central level or at a state/district level? If at the state district level, what made those states adopt the NASG and not others?

9. At what level of the health care system are the NASGs being used?

-Primary? Secondary? Tertiary? Ambulances?

10. If they are used on ambulances, what percent carry the NASG?

-How many NASGs do they carry?

-Who applies the NASG? For example, does the driver or a specially trained EMT apply the NASG?

11. Who is purchasing/providing NASGs in your country?

12. How is the NASG being incorporated into other trainings? For example, is it being integrated into Emergency Obstetric and Newborn Care or Life Saving Skills?

13. Is the NASG being incorporated into the curriculum of clinicians?

-Is it in the Midwifery training? Medical schools? Nursing?

-If so, what are the names of the institutions and when, where, how and at what level is the NASG incorporated in pre-service education?

14. Do the health workers using the NASG have sufficient resources to be able to implement its use properly? For example, enough NASGs and proper cleaning tools for its re-use?

15. Are there any major champions in support of the NASG? Either individuals or organizations.

16. If so, who are they?

-Names, Positions, Contact Information.

17. Are there any factors that have facilitated implementation of the NASG?

18. Are there any challenges that you have observed in integrating the NASG into national standards of care? And/or with increasing accessibility to the NASG?

-If so, have there been any solutions and/or what are the next steps to expand usage?

19. How wide spread do you think the NASG should be in your country? And what do you think it would take to get there?

20. Please provide any additional information you think may be helpful.
