# Supplementary material for: Barriers and Facilitators to Scaling Up the Non-Pneumatic Anti-Shock Garment for Treating Obstetric Hemorrhage: A Qualitative Study
Source: PLoS One. 2016 Mar 3;11(3):e0150739. doi: 10.1371/journal.pone.0150739 (PMC4777561; doi:10.1371/journal.pone.0150739)
Supplement: S2 Appendix — (DOCX) [file pone.0150739.s002.docx]

**S2 Appendix. Basic Demographic Information on Each Key Informant**

**Key Country Current Position Experience relevant**

**Informant to implementation &**

**(KI) scale-up**

1 Ethiopia NGO Program Manager Experience

implementing and scaling-up interventions in multiple low-recourse countries

2 Ethiopia NGO Maternal & Child Experience implementing and scaling-

Health Program Manager up maternal health interventions

in Ethiopia

3 India NGO Program Manager Experience implementing and

scaling-up in pilot project States

4 India NGO Maternal & Child Experience implementing and scaling-

Health Program Director up maternal health interventions in India

5 India WHO Program Manager Previous work with NGO on

implementation and scale-up

6 Nigeria Population & Reproductive Previous work with NGO on

Health Consultant implementation and scale-up

7 Nigeria Academic leadership position Researcher and provider for maternal

at teaching hospital health interventions

8 Nigeria NGO Country Representative Experience implementing and scaling-

up maternal health interventions

9 Nigeria Academic leadership position Researcher and provider for maternal

health interventions

10 Zimbabwe Academic leadership position Researcher and provider for maternal

health interventions

11 Zimbabwe Academic leadership position Researcher and provider for maternal

health interventions

12 Zimbabwe WHO Program Director Implementation science researcher

13 Zimbabwe Maternal Health Project Field researcher for maternal health

Coordinator interventions
