# Supplementary material for: Barriers and Facilitators to Scaling Up the Non-Pneumatic Anti-Shock Garment for Treating Obstetric Hemorrhage: A Qualitative Study
Source: PLoS One. 2016 Mar 3;11(3):e0150739. doi: 10.1371/journal.pone.0150739 (PMC4777561; doi:10.1371/journal.pone.0150739)
Supplement: S3 Appendix — (DOCX) [file pone.0150739.s003.docx]

**S3 Appendix. Code Book**

Attributes of the NASG

-Simplicity

-Acceptance by providers

-Midwives

-Nurses

-Doctors

-Scientific trials

-Cost

-Availability

-Lack of availability

Attributes of the implementers

-Champions

-Partnerships

-Trainings

-Acceptance by government

-Local

-National

Delivery strategy

-Distribution

-Resources

-Transportation

-Ambulances

-Exchange/Return

-Expansion

Attributes of the “adopting” community

-Extent of problem

-Shock

-Blood transfusion

-Delays

-Transport to facility

-At facility

-Cultural beliefs

-Empowerment

-Education

The socio-political context

-Political will

-National policy

-Media

The research context

-Facilitators to implementation

-Barriers to implementation
